# Computational evidence for intramolecular hydrogen bonding and nonbonding X···O interactions in 2'-haloflavonols

**DOI:** 10.3762/bjoc.8.12

**Published:** 2012-01-19

**Authors:** Tânia A O Fonseca, Matheus P Freitas, Rodrigo A Cormanich, Teodorico C Ramalho, Cláudio F Tormena, Roberto Rittner

**Affiliations:** 1 Chemistry Department, Federal University of Lavras, CP 3037, 37200-000, Lavras, MG, Brazil; 2 Chemistry Institute, State University of Campinas, CP 6154, 13083-970, Campinas, Brazil

**Keywords:** conformational analysis, 2'-haloflavonols, intramolecular hydrogen bond, nonbonding interactions, theoretical calculations

## Abstract

The conformational isomerism and stereoelectronic interactions present in 2'-haloflavonols were computationally analyzed. On the basis of the quantum theory of atoms in molecules (QTAIM) and natural bond orbital (NBO) analysis, the conformer stabilities of 2'-haloflavonols were found to be dictated mainly by a C=O···H–O intramolecular hydrogen bond, but an unusual C–F···H–O hydrogen-bond and intramolecular C–X···O nonbonding interactions are also present in such compounds.

## Introduction

Intermolecular hydrogen bonding (HB) is an interaction governing self-assembly and is responsible for the architecture and organization of molecular aggregates [1], and also ligand–receptor interactions that are responsible for the bioactivity of compounds [2]. Moreover, intramolecular HB has been found to govern the conformational preference of molecules [3]. Intramolecular HB involving halogens (X) is less common than those involving oxygen or nitrogen as proton acceptors, while fluorine when bonded to carbon hardly ever participates in HB [4]. Even more unusual stabilizing interactions are the nonbonding F···O interactions, which were experimentally and theoretically characterized in anthracene derivatives, and were pointed out to be the responsible interactions behind the unusual “through-space” fluorine–fluorine spin–spin coupling in the F···O···F fragment present in such a molecular system [5–6]. Although not completely understood, nonbonding F···O interactions, as well as many other unusual long-range interactions involving halogen atoms, are found in several molecular systems in the literature [7–9]. Such weak interactions are more ubiquitous than one imagines and can determine crystal structures [10] and the binding of biological molecules [11] and may possibly be the main forces in determining conformational preferences in molecular systems.

2'-Haloflavonols are important compounds with widespread use as bioactive molecules (antioxidant, anti-inflammatory, antiviral, etc.) [12]. The goal of this work is to understand the intramolecular forces determining the preferred conformations of these molecules through the use of DFT theoretical calculations, quantum theory of atoms in molecules (QTAIM) [13–17] and natural bond orbital (NBO) [18] methods. Possible intramolecular HB and nonbonding F···O interactions are narrowly analyzed and the possible effects of such long-range interactions in determining the rotational isomerism of 2'-haloflavonols are discussed.

## Results and Discussion

**Figure 1:**
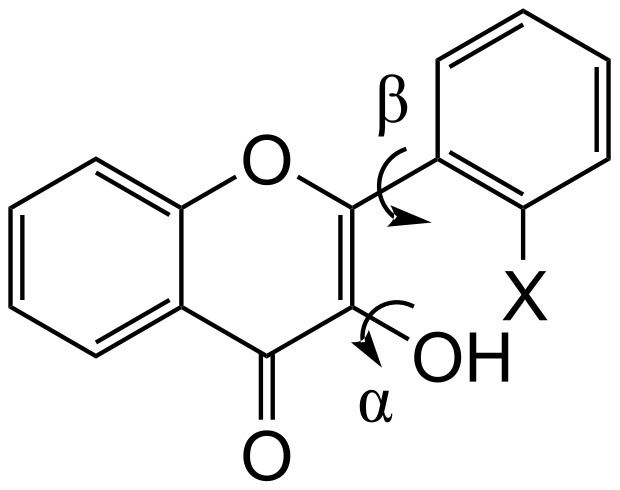
2'-Haloflavonols and the α and β torsional angles.

2'-Haloflavonols undergo rotational isomerization around the α [H–O–C–C(=O)] and β [C(X)–C–C–C(OH)] torsional angles (Figure 1), giving the energy minima obtained at the B3LYP/aug-cc-pVDZ level depicted in Table 1. Flavonol itself (X = H) exhibits two stable conformers, with the most stable one having the hydroxy hydrogen directed toward the carbonyl oxygen (conformer A), establishing an intramolecular HB as the stabilizing interaction of this conformation, in agreement with the crystal structure of 2'-methoxyflavonol [19] and with the bioactive conformation of fisetin [20] and quercetin [21]. Moreover, a weak (H)O···H–C HB also takes place in flavonol (see Supporting Information File 1). NBO analysis at the B3LYP/aug-cc-pVDZ level gives the hyperconjugation contribution for this interaction (*n*_(C=)O_ → σ*_OH_) as 6.9 kcal mol^−1^, while the QTAIM data confirms the establishment of intramolecular HB as a stable, electrostatic interaction (see below).

**Table 1:** Conformational energies (kcal mol^−1^), geometrical parameters (α and β torsional angles in degrees, and C–X distance in angstroms), and selected NBO electron delocalization (kcal mol^−1^) for 2'-haloflavonols.

X	Conf.	E_rel_	α	β	d_C−X_	*n*_O(=C)_/σ*_OH_	*n*_X_/σ*_OH_	*n*_X_/π*_C1C2 + C1C6_

H	A	0	0.0	0.0	1.08	0.8 + 6.1	—	—
	D	9.8	168.5	315.4	1.08	—	—	—
F	A	0	1.1	220.0	1.35	0.6 + 5.0	—	19.2 + 6.0
	B	0.5	2.0	49.3	1.35	0.5 + 4.4	—	19.7 + 6.4
	C	7.8	150.7	43.0	1.37	—	1.1 + 2.6 + 4.6	15.7 + 6.3
	D	8.5	172.9	121.4	1.35	—	—	19.7 + 6.3
Cl	A	0	0.9	232.0	1.76	0.6 + 4.6	—	13.0 + 3.3
	D	7.9	174.8	114.0	1.75	—	—	5.0 + 13.0
Br	A	0	0.9	234.0	1.92	0.5 + 4.6	—	10.2 + 2.4
	D	7.8	175.5	110.7	1.91	—	—	4.0 + 10.1

Among the QTAIM descriptors, the Popelier [17] criteria are useful for the detection and characterization of HB’s, as employed here. The first Popelier criterion is the formation of a bond path between the atoms involved in HB. However, speculation about whether the bond paths obtained from QTAIM may represent steric interactions has been the topic of several discussions in the literature [22–23]. Bader gave special attention to such a question and considered it as a misinterpretation from the QTAIM and physics itself. Indeed, Bader showed that the presence of a bond path linking a pair of atoms fulfills the sufficient and necessary conditions that the atoms are bonded to one another; therefore, the presence of a bond path (together with a BCP and an interatomic surface) always indicates an attractive interaction between two atoms [24–26].

**Figure 2:**
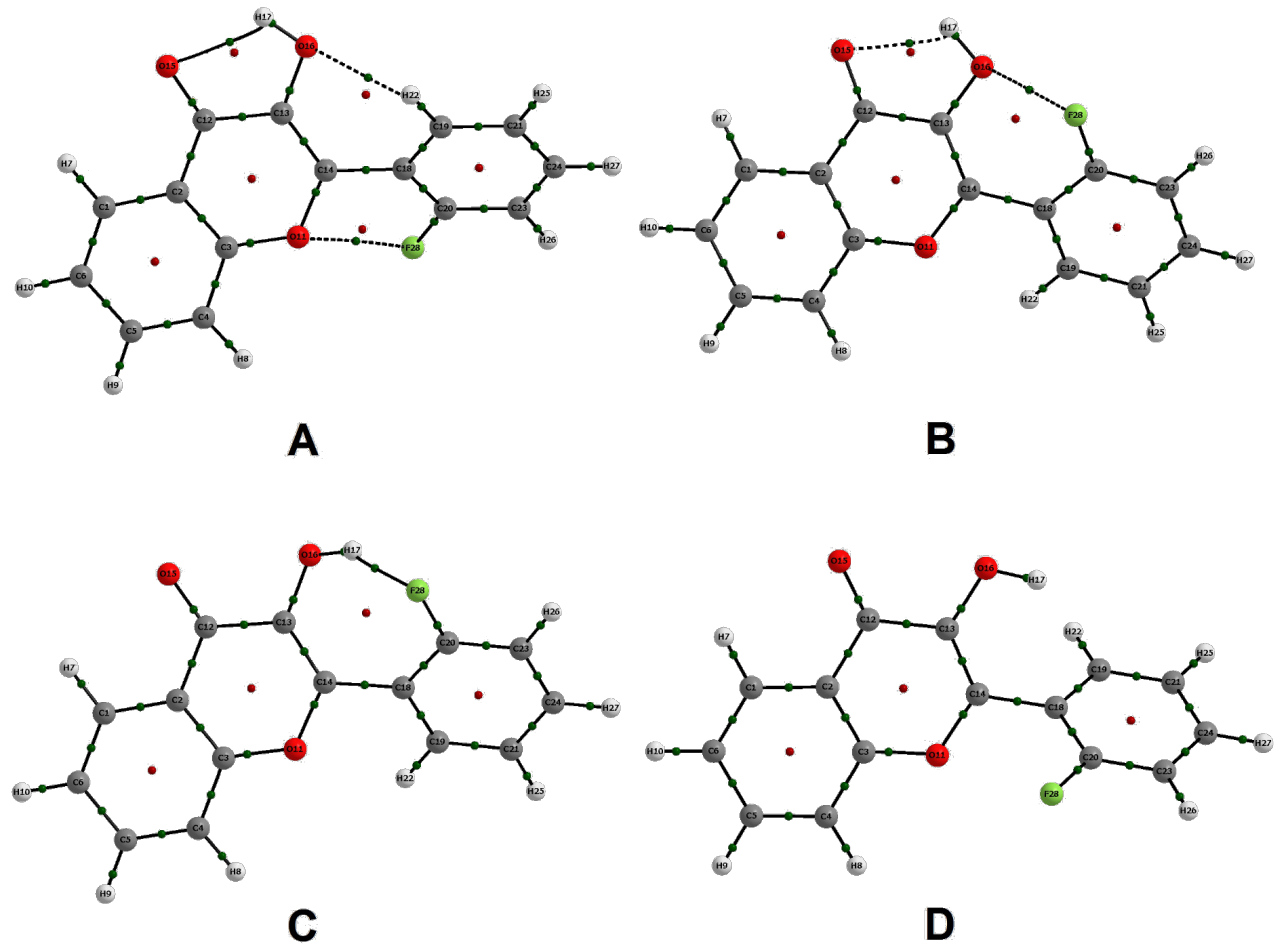
Stable conformers of 2'-fluoroflavonol.

According to Table 2, both the Laplacian of the electronic density (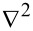
ρ) and the total energy (*H*_C_*)* at the HB bond critical point (BCP) [27] are positive, and the |*V*_C_|/*G*_C_ ratio [28] (where, *V*_C_ = potential energy and *G*_C_ = kinetic energy values at the critical points) is smaller than 1 for the HB’s, indicating the electrostatic character of such interactions, except for the (C=)O···H(O) interaction in 2'-fluoroflavonol A, which is slightly covalent and, hence, stronger than the remaining ones. The distances between the atoms involved in long-range interactions depicted in Table 2 are always shorter than the sum of their van der Waals radii (the van der Waals radii are tabulated in [29]), i.e., such a geometric parameter is fulfilled by all of these interactions, indicating the possibility of their formation [30]. Indeed, when considering the Popelier criteria, the HB’s depicted in Figure 2 and Table 2 are stable and, hence, affect the conformational preferences of the flavonols under study, except for the C^…^H(O) interaction of flavonol B (Table 3). The boldface values in the Table 3 are the reference atoms, i.e., hydrogen atoms that have smaller *q*(H), *E*(H), M_1_(H) and *V*(H) than the reference establish a stable HB.

**Table 2:** QTAIM parameters^a^ (a.u.) and O/F/C···H/X distance^b^ (Å) obtained for selected interacting atoms.

X (Conformer)	O/F/C···H/X	ρ_BCP_	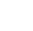 ρ_BCP_	*V*_C_	*G*_C_	*H*_C_	|*V*_C_|/*G*_C_

**H (A)**							
(C=)O···H(O)	1.981	0.028	+0.108	−0.0232	−0.0232	0.0019	0.9243
(C=)O···H(C)	2.647	0.018	+0.074	−0.0124	−0.0124	0.0030	0.8052
**H (B)**							
C···H(O)	2.431	0.013	+0.045	−0.0008	−0.0008	0.0002	0.8889
**F (A)**							
(C=)O···H(O)	2.031	0.027	+0.091	−0.0229	−0.0229	−0.0001	1.0044
(H)O···H(C)	2.499	0.010	+0.041	−0.0071	−0.0071	0.0016	0.8161
(C)O(C)···F	2.703	0.012	+0.054	−0.0112	−0.0112	0.0011	0.9106
**F (B)**							
(C=)O···H(O)	2.057	0.025	+0.099	−0.0199	−0.0199	0.0024	0.8924
(H)O···F	2.794	0.010	+0.042	−0.0087	−0.0087	0.0009	0.9063
**F (C)**							
F···H(O)	1.853	0.028	+0.102	−0.0247	−0.0247	0.0005	0.9841
**Cl (A)**							
(C=)O···H(O)	2.047	0.025	+0.099	−0.0202	−0.0202	0.0023	0.8978
(C)O(C)···Cl	3.069	0.011	+0.043	−0.0076	−0.0076	0.0016	0.8352
**Br (A)**							
(C=)O···H(O)	2.050	0.025	+0.099	−0.0201	−0.0201	0.0023	0.8973
(C)O(C)···Br	3.189	0.010	+0.038	−0.0070	−0.0070	0.0013	0.8537

^a^ρ_BCP_ = electronic density along with BCP; 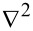
ρ_BCP_ = Laplacian of the electronic density along with BCP. ^b^O/F/C···H/X = distance between long-range interacting oxygen/fluorine/carbon and hydrogen/halogen atoms.

**Table 3:** QTAIM parameters (a.u.) for the hydrogen involved in HB and the halogens involved in electrostatic halogen bonding.

X (Conformer)	*q*(Ω)	*E*(Ω)	M_1_(Ω)	*V*(Ω)

**H (A)**				
H(O)	+0.601	−0.356	0.145	17.921
H(C)	+0.082	−0.599	0.113	39.732
**H (B)**				
H(O)	+0.567	−0.379	0.165	21.108
H(C)	**+0.045**	**−0.606**	**0.134**	**46.738**
**F (A)**				
H(O)	+0.620	−0.331	0.151	17.809
H(C)	+0.043	−0.605	0.121	44.374
**F (B)**				
H(O)	+0.599	−0.358	0.148	18.626
**F (C)**				
H(O)	+0.623	−0.341	0.131	14.335
**F (D)**				
H(O)	**+0.567**	**−0.379**	**0.165**	**21.744**
H(C)	**+0.047**	**−0.604**	**0.134**	**47.458**
**Cl (A)**				
H(O)	+0.598	−0.358	0.148	18.584
**Cl (B)**				
H(O)	**+0.567**	**−0.379**	**0.165**	**22.029**
**Br (A)**				
H(O)	+0.598	−0.358	0.148	18.584
**Br (B)**				
H(O)	**+0.567**	**−0.379**	**0.165**	**22.029**

2'-Fluoroflavonol exhibits four stable conformers, in which those with the hydroxy hydrogen directed toward the carbonyl oxygen (A and B) are significantly more stable than the other two conformers (Table 1); clearly, the intramolecular HB O–H···O=C plays the determinant role for the conformational isomerism of 2'-fluoroflavonol, as confirmed by NBO (*n*_O(=C)_ → σ*_OH_ ≥ 5 kcal mol^−1^) and QTAIM calculations (Table 1 and Table 2). The most stable form (A, Figure 2) has the hydroxy oxygen far from the fluorine atom bonded to the adjacent phenyl ring and, according to QTAIM data, it establishes two other stabilizing interactions: Weak HB H–O···H–C and a nonbonding F^…^O interaction. The formation of the nonbonding F^…^O interaction in conformer A, having fluorine as electron acceptor, is supposed to be due to the partial negative atomic charge on the ether oxygen and a less negative atomic charge on the fluorine atom (the QTAIM atomic charges are −0.619 for F and −1.075 for O). Conformer B, in which fluorine is close to the hydroxy oxygen, is calculated to be 0.5 kcal mol^−1^ less stable than A. According to the classical sense, this energy difference would be attributed to dipolar repulsion between the polar bonds; however, the QTAIM calculations indicate that the interaction between fluorine and oxygen is attractive, suggesting the establishment of a nonbonding F^…^O interaction of similar magnitude to that found in conformer A (see the electronic density along with BCP and the Laplacian values in Table 2). In this case, the fluorine atom is the electron acceptor (QTAIM atomic charge of −0.620 for F against −1.093 for the hydroxy oxygen), which is not so uncommon, as stated in classical textbooks on aromatic electrophilic substitution, since a resonant structure with a C=F^+^ contribution can take place [31]; this is shown by NBO calculations, which indicate an interaction responsible for this resonance (*n*_F_ → π*_C1C2_) of ca. 20 kcal mol^−1^ (Table 1). Therefore, the difference in stability between A and B can be estimated to be due to the weak HB H–O···H–C together with the slightly covalent character of C=O···H–O in A.

Moreover, conformer C is predicted to be more stable than D by 0.7 kcal mol^−1^. Again, the basic difference between them is the orientation of the fluorine atom, in which C experiences intramolecular HB O–H···F–C according to the electron delocalizations obtained by NBO (sum of *n*_F_ → σ*_OH_ = 8.2 kcal mol^−1^), as well as by QTAIM results (Table 1 and Table 2). There is not an attractive interaction observed for D by means of QTAIM calculations; therefore, the energy difference between C and D is expected to be due to the intramolecular HB O–H···F–C. Furthermore, the longer C–X distance and weaker *n*_F_ → π*_CC_ electron delocalization in C than in the remaining conformers (Table 1) provide evidence that fluorine lone pairs in C are involved in intramolecular HB O–H···F–C instead of contributing to the resonant structure with C=F^+^. HB involving fluorine as a proton acceptor when bonded to carbon is unusual [4], but it has been shown to be of secondary, not negligible, importance for the conformational isomerism of 2'-fluoroflavonol in this work.

The conformational behaviours of chlorine and bromine derivatives are quite similar to each other, given the energy difference between the two stable conformers and their geometrical parameters (Table 1 and Supporting Information File 1). According to QTAIM calculations, conformer A of both 2'-haloflavonols experiences intramolecular HB C=O···H–O and, to a lesser extent, a nonbonding C–X···O–C interaction, while conformer D, in which the halogen is far from the hydroxy group and the hydroxy hydrogen is not directed toward the carbonyl oxygen, does not exhibit any attractive interaction like these ones. Again, the conformational isomerism of 2'-chloro and 2'-bromoflavonol is governed by the intramolecular HB C=O···H–O (see NBO *n*_O(=C)_/σ*_OH_ electron delocalizations and QTAIM C=O/HO electron densities and Laplacian in Table 1 and Table 2), but a nonbonding C–X···O–C interaction also operates in A. The nonbonding C–X···O–C interaction in chlorine and bromine derivatives is expected to be inferior to that in 2'-fluoroflavonol, given the lower *n*_X_/π*_CC_ delocalization energies, making the contribution of C=Cl^+^ and C=Br^+^ less significant for the resonance structure; also, both intramolecular HB and nonbonding C–X···O–C interactions with OH are impossible for the chlorine and bromine derivatives, since the halogen in the optimized conformers A and D are not directed toward OH.

## Conclusion

Overall, the intramolecular HB C=O···H–O is the dictating force of the conformational equilibrium of 2'-haloflavonols, but a nonbonding C–X···O–C interaction is also operating. Other intramolecular HBs are experienced by 2'-fluoroflavonol, including in particular an unusual C–F···H–O interaction. In fact, the strong resonant structure due to the C–X···O–C interaction in *ortho* position to a benzene ring conjugated with an α,β-unsaturated ketone is supposed to favour the C=X^+^ contribution and, therefore, to establish nonbonding C–X^…^O–C interactions, in addition to intramolecular HB, especially for the fluorine derivative. Thus, our findings suggest that nonbonding C–X···O–C interactions may be a driving force toward the bioactive conformation of molecules.

## Supporting Information

File 1 Optimized structures for all minima of 2'-haloflavonols and the corresponding Cartesian coordinates.
