# Supplementary material for: Computational evidence for intramolecular hydrogen bonding and nonbonding X···O interactions in 2'-haloflavonols
Source: Beilstein J Org Chem. 2012 Jan 19;8:112–7. doi: 10.3762/bjoc.8.12 (PMC3302071; doi:10.3762/bjoc.8.12)
Supplement: File 1 — Optimized structures for all minima of 2'-haloflavonols and the corresponding Cartesian coordinates. [file Beilstein_J_Org_Chem-08-112-s001.pdf]

Supporting Information

for

Computational evidence for intramolecular hydrogen bonding and nonbonding X···O interactions in 2'-haloflavonols

Tânia A. O. Fonseca[1], Matheus P. Freitas[*1], Rodrigo A. Cormanich[2], Teodorico C. Ramalho[1], Cláudio F. Tormena[2], and Roberto Rittner[2]

Address: [1]Chemistry Department, Federal University of Lavras, CP 3037, 37200-000, Lavras, MG, Brazil and [2]Chemistry Institute, State University of Campinas, CP 6154, 13083-970, Campinas, Brazil.

Email: Matheus P. Freitas - matheus@dqi.ufla.br

*Corresponding author

Optimized structures for all minima of 2'-haloflavonols and the corresponding Cartesian coordinates

Table of Contents

Page S3. Stable conformers for the 2’-X-flavonols (X = H, F, Cl and Br).

Page S4. Final structure of flavonol (conformer A) in terms of initial Z-matrix.

Page S6. Final structure of flavonol (conformer B) in terms of initial Z-matrix.

Page S8. Final structure of 2'-fluoroflavonol (conformer A) in terms of initial Z-matrix.

Page S10. Final structure of 2'-fluoroflavonol (conformer B) in terms of initial Z-matrix.

Page S12. Final structure of 2'-fluoroflavonol (conformer C) in terms of initial Z-matrix.

Page S14. Final structure of 2'-fluoroflavonol (conformer D) in terms of initial Z-matrix.

Page S16. Final structure of 2'-chloroflavonol (conformer A) in terms of initial Z-matrix.

Page S18. Final structure of 2'-chloroflavonol (conformer D) in terms of initial Z-matrix.

Page S20. Final structure of 2'-bromoflavonol (conformer A) in terms of initial Z-matrix.

Page S22. Final structure of 2'-bromoflavonol (conformer D) in terms of initial Z-matrix.

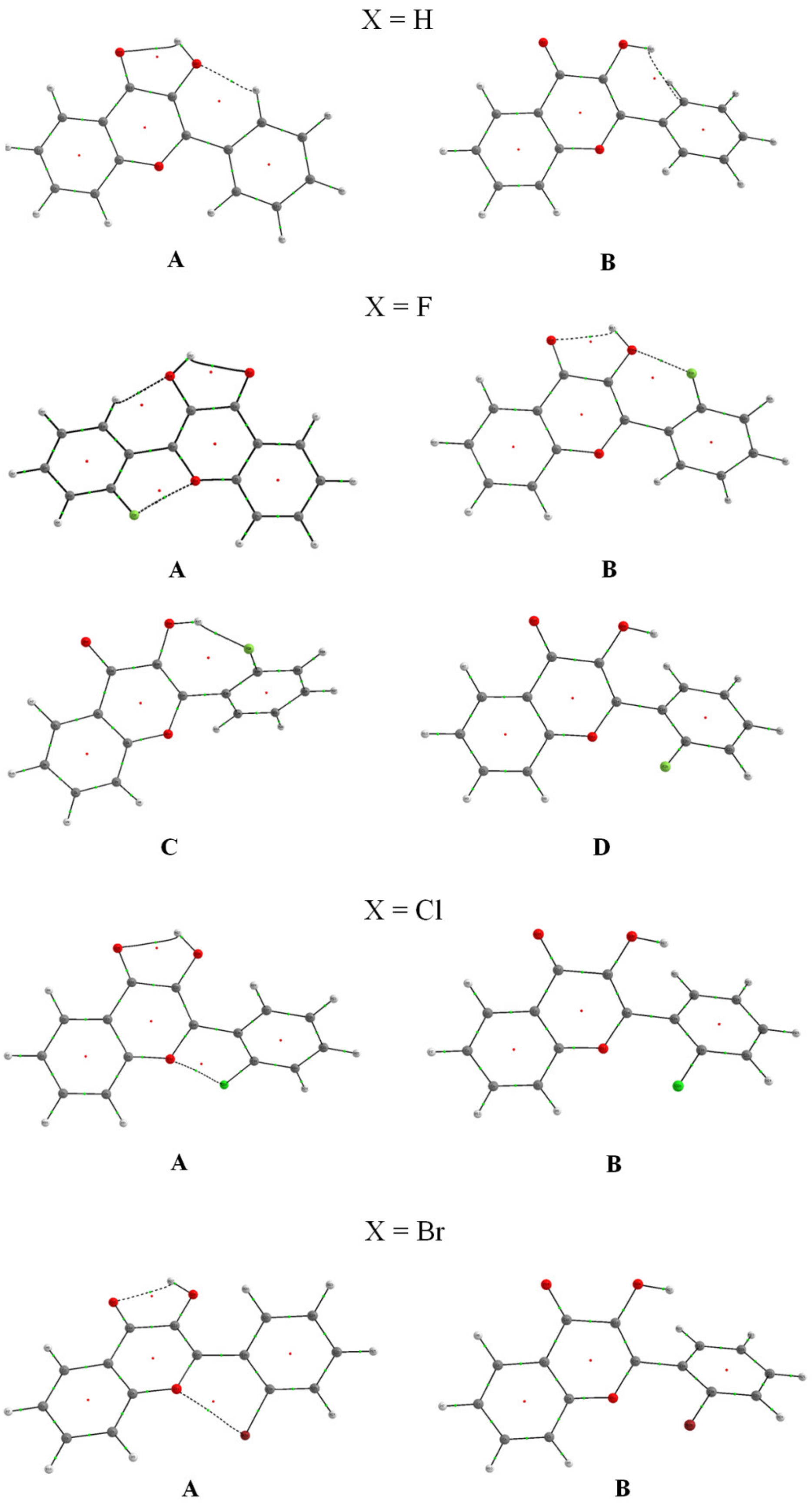

S1. Stable conformers for the 2'-X-flavonols (X = H, F, Cl and Br).

S2. Optimized structures for 2'-X-flavonols:

X = H

Conformer A

Final structure in terms of initial Z-matrix:
C
C,1,B1
C,2,B2,1,A1
C,3,B3,2,A2,1,D1,0
C,4,B4,3,A3,2,D2,0
C,5,B5,4,A4,3,D3,0
H,1,B6,2,A5,3,D4,0
H,2,B7,1,A6,6,D5,0
H,5,B8,4,A7,3,D6,0
H,6,B9,5,A8,4,D7,0
O,4,B10,3,A9,2,D8,0
C,3,B11,2,A10,1,D9,0
C,12,B12,3,A11,2,D10,0
C,13,B13,12,A12,3,D11,0
O,12,B14,3,A13,2,D12,0
C,14,B15,13,A14,12,D13,0
C,16,B16,14,A15,13,D14,0
C,16,B17,14,A16,13,D15,0
C,17,B18,16,A17,14,D16,0
H,17,B19,16,A18,14,D17,0
C,18,B20,16,A19,14,D18,0
H,18,B21,16,A20,14,D19,0
C,21,B22,18,A21,16,D20,0
H,19,B23,17,A22,16,D21,0
H,21,B24,18,A23,16,D22,0
H,23,B25,21,A24,18,D23,0
O,13,B26,12,A25,3,D24,0
H,27,B27,13,A26,12,D25,0
Variables:
B1=1.38116693
B2=1.40703418
B3=1.40025312
B4=1.39949105
B5=1.38373876
B6=1.08336361
B7=1.08321129
B8=1.08290359
B9=1.08398138
B10=1.35806881
B11=1.45562765
B12=1.45961276
B13=1.36744807
B14=1.23701555
B15=1.46810621
B16=1.40808849
B17=1.40782297
B18=1.38935293
B19=1.08084534
B20=1.39120169
B21=1.07885843
B22=1.39318453
B23=1.08421473
B24=1.08412969
B25=1.08412392
B26=1.35255706
B27=0.97927312
A1=120.25402114
A2=118.96880778
A3=121.11615706

A4=118.88121459
A5=120.25099798
A6=121.77880056
A7=119.28715259
A8=119.35585371
A9=121.98105292
A10=122.5647566
A11=115.89453525
A12=122.37309043
A13=125.46150417
A14=128.60829376
A15=119.694646
A16=121.92328323
A17=120.75499977
A18=119.58094557
A19=120.37271665
A20=119.92075696
A21=120.71246104
A22=119.5015909
A23=119.22531728
A24=120.32464275
A25=114.04175915
A26=104.01454434
D1=-0.00220703
D2=0.00294222
D3=-0.00216584
D4=-179.99856675
D5=179.99988276
D6=179.99757097
D7=179.9999285
D8=-179.99929166
D9=179.99562653
D10=-179.98803548
D11=-0.02903441
D12=0.01213897
D13=-179.96876076
D14=179.93217436
D15=-0.07154779
D16=-179.99798178
D17=-0.00373198
D18=179.99856924
D19=-0.00375349
D20=0.00110149
D21=179.99828314
D22=-179.99782877
D23=-179.99958033
D24=179.98860175
D25=0.00966111

Conformer B

Final structure in terms of initial Z-matrix:
C
C,1,B1
C,2,B2,1,A1
C,3,B3,2,A2,1,D1,0
C,4,B4,3,A3,2,D2,0
C,5,B5,4,A4,3,D3,0
H,1,B6,2,A5,3,D4,0
H,2,B7,1,A6,6,D5,0
H,5,B8,4,A7,3,D6,0
H,6,B9,5,A8,4,D7,0
O,4,B10,3,A9,2,D8,0
C,3,B11,2,A10,1,D9,0
C,12,B12,3,A11,2,D10,0
C,13,B13,12,A12,3,D11,0
O,12,B14,3,A13,2,D12,0
C,14,B15,13,A14,12,D13,0
C,16,B16,14,A15,13,D14,0
C,16,B17,14,A16,13,D15,0
C,17,B18,16,A17,14,D16,0
H,17,B19,16,A18,14,D17,0
C,18,B20,16,A19,14,D18,0
H,18,B21,16,A20,14,D19,0
C,21,B22,18,A21,16,D20,0
H,19,B23,17,A22,16,D21,0
H,21,B24,18,A23,16,D22,0
H,23,B25,21,A24,18,D23,0
O,13,B26,12,A25,3,D24,0
H,27,B27,13,A26,12,D25,0
Variables:
B1=1.38233893
B2=1.4059077
B3=1.39802672
B4=1.39871463
B5=1.38432111
B6=1.0834752
B7=1.08328717
B8=1.08301566
B9=1.08401249
B10=1.36349337
B11=1.47509212
B12=1.47226595
B13=1.36252391
B14=1.22227611
B15=1.47570937
B16=1.40413432
B17=1.40353536
B18=1.39323155
B19=1.08429178
B20=1.39043015
B21=1.08295438
B22=1.39519344
B23=1.08394182
B24=1.0840604
B25=1.08388964
B26=1.3596579
B27=0.96513194
A1=120.54308429
A2=118.50147701
A3=121.52669292
A4=118.8421706
A5=120.20805404
A6=121.80577291
A7=119.25402256
A8=119.43136061
A9=121.70951345
A10=121.10569104
A11=113.89499172
A12=121.55634731

A13=123.43936127
A14=125.76204341
A15=121.08414055
A16=120.06956677
A17=120.45566128
A18=119.9536916
A19=120.4358136
A20=119.36751651
A21=120.33740169
A22=119.59396281
A23=119.63924092
A24=120.14938572
A25=114.55831158
A26=109.94002008
D1=-0.08800948
D2=0.09635249
D3=-0.05922609
D4=-179.94934329
D5=-179.84660385
D6=179.72395581
D7=179.94233798
D8=179.66032551
D9=-179.2145768
D10=179.27066144
D11=2.12694214
D12=-0.15498182
D13=179.37003567
D14=-44.57574492
D15=136.18944471
D16=179.63745079
D17=-2.96485214
D18=-179.64516585
D19=0.1563781
D20=-0.26623349
D21=179.44500549
D22=179.58666864
D23=179.96559541
D24=-177.35644831
D25=168.61603958

X = F

Conformer A

Final structure in terms of initial Z-matrix:
C
C,1,B1
C,2,B2,1,A1
C,3,B3,2,A2,1,D1,0
C,4,B4,3,A3,2,D2,0
C,1,B5,2,A4,3,D3,0
H,1,B6,6,A5,5,D4,0
H,4,B7,3,A6,2,D5,0
H,5,B8,4,A7,3,D6,0
H,6,B9,1,A8,2,D7,0
O,3,B10,2,A9,1,D8,0
C,2,B11,1,A10,6,D9,0
C,12,B12,2,A11,1,D10,0
C,13,B13,12,A12,2,D11,0
O,12,B14,2,A13,1,D12,0
O,13,B15,12,A14,2,D13,0
H,16,B16,13,A15,12,D14,0
C,14,B17,13,A16,12,D15,0
C,18,B18,14,A17,13,D16,0
C,18,B19,14,A18,13,D17,0
C,19,B20,18,A19,14,D18,0
H,19,B21,18,A20,14,D19,0
C,20,B22,18,A21,14,D20,0
C,23,B23,20,A22,18,D21,0
H,21,B24,19,A23,18,D22,0
H,23,B25,20,A24,18,D23,0
H,24,B26,23,A25,20,D24,0
F,20,B27,18,A26,14,D25,0
Variables:
B1=1.40759653
B2=1.40204224
B3=1.39951865
B4=1.38318459
B5=1.38075606
B6=1.08319638
B7=1.08275271
B8=1.08397494
B9=1.08338757
B10=1.3586117
B11=1.45733994
B12=1.46000771
B13=1.36170075
B14=1.23578592
B15=1.35241875
B16=0.97738951
B17=1.47004062
B18=1.40476859
B19=1.40045527
B20=1.38956148
B21=1.0815568
B22=1.3850104
B23=1.39208637
B24=1.08324285
B25=1.08284683
B26=1.08367301
B27=1.35207956
A1=118.83556492
A2=121.19611459
A3=118.85026555
A4=120.29320683
A5=121.77750712
A6=119.11524807

A7=119.36582758
A8=120.22746403
A9=122.06523099
A10=122.30462338
A11=115.42487666
A12=121.92141341
A13=125.67591027
A14=115.26749093
A15=104.34442218
A16=125.97406825
A17=120.8620474
A18=122.17632986
A19=121.25263946
A20=118.6903306
A21=122.65840356
A22=119.07083319
A23=119.67032537
A24=118.98495142
A25=119.64038522
A26=119.6856544
D1=0.04846074
D2=0.00819975
D3=-0.08046305
D4=-179.91327343
D5=-179.82725351
D6=-179.94617704
D7=-179.980777
D8=179.75975362
D9=-179.89263687
D10=179.7665228
D11=-0.31870042
D12=0.45642484
D13=-179.21397538
D14=1.0884314
D15=-179.65050727
D16=-40.16881133
D17=139.94517422
D18=-179.09370519
D19=0.9209714
D20=179.07102497
D21=0.25940026
D22=179.62445343
D23=179.65697226
D24=179.96026948
D25=-2.6053541

Conformer B

Final structure in terms of initial Z-matrix:
C
C,1,B1
C,2,B2,1,A1
C,3,B3,2,A2,1,D1,0
C,4,B4,3,A3,2,D2,0
C,1,B5,2,A4,3,D3,0
H,1,B6,6,A5,5,D4,0
H,4,B7,3,A6,2,D5,0
H,5,B8,4,A7,3,D6,0
H,6,B9,1,A8,2,D7,0
O,3,B10,2,A9,1,D8,0
C,2,B11,1,A10,6,D9,0
C,12,B12,2,A11,1,D10,0
C,13,B13,12,A12,2,D11,0
O,12,B14,2,A13,1,D12,0
O,13,B15,12,A14,2,D13,0
H,16,B16,13,A15,12,D14,0
C,14,B17,13,A16,12,D15,0
C,18,B18,14,A17,13,D16,0
C,18,B19,14,A18,13,D17,0
C,19,B20,18,A19,14,D18,0
H,19,B21,18,A20,14,D19,0
C,20,B22,18,A21,14,D20,0
C,23,B23,20,A22,18,D21,0
H,21,B24,19,A23,18,D22,0
H,23,B25,20,A24,18,D23,0
H,24,B26,23,A25,20,D24,0
F,20,B27,18,A26,14,D25,0
Variables:
B1=1.40739781
B2=1.4017715
B3=1.3994726
B4=1.38351657
B5=1.38096023
B6=1.08323939
B7=1.08291619
B8=1.08396978
B9=1.0833759
B10=1.36067555
B11=1.45901531
B12=1.46135244
B13=1.35975468
B14=1.23439964
B15=1.34756467
B16=0.9759152
B17=1.46991948
B18=1.40461583
B19=1.39757137
B20=1.38897271
B21=1.08309821
B22=1.38655852
B23=1.39129381
B24=1.08333406
B25=1.0827999
B26=1.08368873
B27=1.3475099
A1=118.7950312
A2=121.18876971
A3=118.89419507
A4=120.35605395
A5=121.75560066
A6=119.19376974
A7=119.37332633
A8=120.23437875
A9=122.00691458
A10=122.13474909
A11=115.38325745
A12=121.74520301

A13=125.49023034
A14=115.76104829
A15=104.85437273
A16=126.5831262
A17=120.38190791
A18=122.39867746
A19=121.22603955
A20=118.56939764
A21=122.45854212
A22=119.08244699
A23=119.8502374
A24=118.99330316
A25=119.62146948
A26=119.52139073
D1=-0.06719913
D2=-0.01698537
D3=0.12026119
D4=179.87713023
D5=-179.77034661
D6=-179.89126212
D7=179.90569094
D8=-179.71781144
D9=179.42657835
D10=-179.85645328
D11=-0.9537714
D12=-0.77726088
D13=177.43168802
D14=1.99547077
D15=178.58098308
D16=-131.43490456
D17=49.27195578
D18=179.07788463
D19=-0.17401814
D20=-178.91275759
D21=-0.74394264
D22=-179.27625199
D23=-179.93805005
D24=179.9400511
D25=3.34286074

Conformer C

Final structure in terms of initial Z-matrix:
C
C,1,B1
C,2,B2,1,A1
C,3,B3,2,A2,1,D1,0
C,4,B4,3,A3,2,D2,0
C,1,B5,2,A4,3,D3,0
H,1,B6,6,A5,5,D4,0
H,4,B7,3,A6,2,D5,0
H,5,B8,4,A7,3,D6,0
H,6,B9,1,A8,2,D7,0
O,3,B10,2,A9,1,D8,0
C,2,B11,1,A10,6,D9,0
C,12,B12,2,A11,1,D10,0
C,13,B13,12,A12,2,D11,0
O,12,B14,2,A13,1,D12,0
O,13,B15,12,A14,2,D13,0
H,16,B16,13,A15,12,D14,0
C,14,B17,13,A16,12,D15,0
C,18,B18,14,A17,13,D16,0
C,18,B19,14,A18,13,D17,0
C,19,B20,18,A19,14,D18,0
H,19,B21,18,A20,14,D19,0
C,20,B22,18,A21,14,D20,0
C,23,B23,20,A22,18,D21,0
H,21,B24,19,A23,18,D22,0
H,23,B25,20,A24,18,D23,0
H,24,B26,23,A25,20,D24,0
F,20,B27,18,A26,14,D25,0
Variables:
B1=1.40582275
B2=1.39638714
B3=1.39892738
B4=1.38435064
B5=1.38232244
B6=1.08327779
B7=1.08303791
B8=1.08403368
B9=1.08345851
B10=1.36242321
B11=1.4736745
B12=1.47941495
B13=1.36469316
B14=1.22108792
B15=1.35363756
B16=0.96443127
B17=1.47687415
B18=1.40819401
B19=1.39474433
B20=1.38811478
B21=1.08202786
B22=1.38373169
B23=1.39080332
B24=1.0833126
B25=1.08264618
B26=1.08333336
B27=1.36906118
A1=118.58131133
A2=121.544946
A3=118.78183862
A4=120.48413552
A5=121.83486457
A6=119.32467641
A7=119.41629691
A8=120.22605069
A9=121.64696755
A10=121.27256663
A11=114.49585674
A12=121.05788991

A13=123.24926833
A14=113.97084283
A15=110.98374094
A16=128.13208962
A17=119.36899268
A18=124.69357208
A19=121.58884621
A20=118.31795366
A21=123.8484643
A22=118.67081158
A23=119.63086313
A24=119.19354762
A25=119.75376114
A26=119.04793851
D1=-0.15521833
D2=0.18021096
D3=0.02953301
D4=-179.91331818
D5=-179.70141681
D6=179.96719726
D7=-179.9844185
D8=-179.9000714
D9=-179.53460628
D10=179.47691093
D11=1.13009992
D12=-0.99631224
D13=177.31520506
D14=150.71390648
D15=173.93092615
D16=-138.19482591
D17=42.99059959
D18=179.90774802
D19=0.26280105
D20=-179.95570922
D21=-0.48811914
D22=-179.35196235
D23=-179.76928344
D24=-179.99728603
D25=1.12251138

Conformer D

Final structure in terms of initial Z-matrix:
C
C,1,B1
C,2,B2,1,A1
C,3,B3,2,A2,1,D1,0
C,4,B4,3,A3,2,D2,0
C,1,B5,2,A4,3,D3,0
H,1,B6,6,A5,5,D4,0
H,4,B7,3,A6,2,D5,0
H,5,B8,4,A7,3,D6,0
H,6,B9,1,A8,2,D7,0
O,3,B10,2,A9,1,D8,0
C,2,B11,1,A10,6,D9,0
C,12,B12,2,A11,1,D10,0
C,13,B13,12,A12,2,D11,0
O,12,B14,2,A13,1,D12,0
O,13,B15,12,A14,2,D13,0
H,16,B16,13,A15,12,D14,0
C,14,B17,13,A16,12,D15,0
C,18,B18,14,A17,13,D16,0
C,18,B19,14,A18,13,D17,0
C,19,B20,18,A19,14,D18,0
H,19,B21,18,A20,14,D19,0
C,20,B22,18,A21,14,D20,0
C,23,B23,20,A22,18,D21,0
H,21,B24,19,A23,18,D22,0
H,23,B25,20,A24,18,D23,0
H,24,B26,23,A25,20,D24,0
F,20,B27,18,A26,14,D25,0
Variables:
B1=1.40625542
B2=1.3989923
B3=1.39864302
B4=1.38397661
B5=1.38202789
B6=1.08325348
B7=1.08289624
B8=1.08400998
B9=1.08347239
B10=1.36326112
B11=1.47503025
B12=1.47357497
B13=1.35903727
B14=1.22173023
B15=1.35896271
B16=0.96519678
B17=1.47851068
B18=1.40397347
B19=1.39797712
B20=1.3920452
B21=1.08419874
B22=1.38524152
B23=1.39313326
B24=1.08315381
B25=1.08285355
B26=1.08359516
B27=1.34930512
A1=118.47278805
A2=121.53381261
A3=118.8293332
A4=120.5433153
A5=121.8093591
A6=119.19248913
A7=119.42298833
A8=120.20435048
A9=121.74874044
A10=120.9838934
A11=113.71026995
A12=121.28456302

A13=123.62618399
A14=114.95947278
A15=109.92061989
A16=124.34339987
A17=121.13484813
A18=121.63007711
A19=121.13541183
A20=118.75902205
A21=122.63079
A22=118.89749443
A23=119.83171557
A24=119.22683828
A25=119.53433694
A26=118.98903373
D1=0.02415587
D2=0.01265412
D3=-0.06326635
D4=-179.87199536
D5=-179.91718346
D6=-179.95174006
D7=-179.97538046
D8=179.53806559
D9=-179.53204595
D10=179.02817132
D11=1.68393923
D12=-0.54581177
D13=-177.92591481
D14=172.89348469
D15=178.43200968
D16=-58.91422552
D17=121.35242583
D18=-179.94362541
D19=-1.29162725
D20=179.63480712
D21=0.25498868
D22=179.61321699
D23=179.83230408
D24=179.96091123
D25=-1.31171269

X = Cl

Conformer A

Final structure in terms of initial Z-matrix:
C
C,1,B1
C,2,B2,1,A1
C,3,B3,2,A2,1,D1,0
C,4,B4,3,A3,2,D2,0
C,5,B5,4,A4,3,D3,0
H,1,B6,2,A5,3,D4,0
H,2,B7,1,A6,6,D5,0
H,5,B8,4,A7,3,D6,0
H,6,B9,5,A8,4,D7,0
O,4,B10,3,A9,2,D8,0
C,3,B11,2,A10,1,D9,0
C,12,B12,3,A11,2,D10,0
C,13,B13,12,A12,3,D11,0
O,12,B14,3,A13,2,D12,0
C,14,B15,13,A14,12,D13,0
C,16,B16,14,A15,13,D14,0
C,16,B17,14,A16,13,D15,0
C,17,B18,16,A17,14,D16,0
H,17,B19,16,A18,14,D17,0
C,18,B20,16,A19,14,D18,0
C,21,B21,18,A20,16,D19,0
H,19,B22,17,A21,16,D20,0
H,21,B23,18,A22,16,D21,0
H,22,B24,21,A23,18,D22,0
O,13,B25,12,A24,3,D23,0
H,26,B26,13,A25,12,D24,0
Cl,18,B27,16,A26,14,D25,0
Variables:
B1=1.38079133
B2=1.40759267
B3=1.40237188
B4=1.39951608
B5=1.38319425
B6=1.08339862
B7=1.08322095
B8=1.08277354
B9=1.08398444
B10=1.35912282
B11=1.45825945
B12=1.4610936
B13=1.35894919
B14=1.23500785
B15=1.47486578
B16=1.40290058
B17=1.40389352
B18=1.38918345
B19=1.08257678
B20=1.39166236
B21=1.39159984
B22=1.08338427
B23=1.08240066
B24=1.08386869
B25=1.3520425
B26=0.97664201
B27=1.75745825
A1=120.31912071
A2=118.79610726
A3=121.21033421
A4=118.85808964
A5=120.22461107
A6=121.7646442

A7=119.11575433
A8=119.3721979
A9=122.03665474
A10=122.22161424
A11=115.29471916
A12=121.80916981
A13=125.68333127
A14=125.42598071
A15=119.05429826
A16=123.13339575
A17=121.33434279
A18=118.5135709
A19=121.21429431
A20=119.78331943
A21=119.84568155
A22=119.37936091
A23=119.48708287
A24=115.60257698
A25=104.5141405
A26=121.09559036
D1=0.07929495
D2=-0.08364174
D3=0.03433602
D4=179.98186594
D5=179.94269452
D6=179.90967668
D7=179.93733442
D8=-179.68211023
D9=179.99620295
D10=-179.72528645
D11=0.39767902
D12=-0.27283264
D13=-179.72305995
D14=52.05102501
D15=-128.02723694
D16=178.77777817
D17=-1.05365212
D18=-178.63008765
D19=-0.48111325
D20=-179.66376608
D21=-179.73725307
D22=-179.96700627
D23=179.35000261
D24=-0.88458511
D25=3.97176791

Conformer D

Final structure in terms of initial Z-matrix:
C
C,1,B1
C,2,B2,1,A1
C,3,B3,2,A2,1,D1,0
C,4,B4,3,A3,2,D2,0
C,5,B5,4,A4,3,D3,0
H,1,B6,2,A5,3,D4,0
H,2,B7,1,A6,6,D5,0
H,5,B8,4,A7,3,D6,0
H,6,B9,5,A8,4,D7,0
O,4,B10,3,A9,2,D8,0
C,3,B11,2,A10,1,D9,0
C,12,B12,3,A11,2,D10,0
C,13,B13,12,A12,3,D11,0
O,12,B14,3,A13,2,D12,0
C,14,B15,13,A14,12,D13,0
C,16,B16,14,A15,13,D14,0
C,16,B17,14,A16,13,D15,0
C,17,B18,16,A17,14,D16,0
H,17,B19,16,A18,14,D17,0
C,18,B20,16,A19,14,D18,0
C,21,B21,18,A20,16,D19,0
H,19,B22,17,A21,16,D20,0
H,21,B23,18,A22,16,D21,0
H,22,B24,21,A23,18,D22,0
O,13,B25,12,A24,3,D23,0
H,26,B26,13,A25,12,D24,0
Cl,18,B27,16,A26,14,D25,0
Variables:
B1=1.38208611
B2=1.40623627
B3=1.39918288
B4=1.39868978
B5=1.38397658
B6=1.08349538
B7=1.08327918
B8=1.08292727
B9=1.08403473
B10=1.36327939
B11=1.47541158
B12=1.47380734
B13=1.35761748
B14=1.2215817
B15=1.48182656
B16=1.40334785
B17=1.40360603
B18=1.39150105
B19=1.08428954
B20=1.3917138
B21=1.39235722
B22=1.08333784
B23=1.08245653
B24=1.08378549
B25=1.35887291
B26=0.96526891
B27=1.75332134
A1=120.55271704
A2=118.45441587
A3=121.5422623
A4=118.83203397
A5=120.20248473
A6=121.79721097
A7=119.18511923
A8=119.42795234
A9=121.73782298
A10=120.97830265
A11=113.66463144
A12=121.26712743

A13=123.63034177
A14=124.11396412
A15=119.56465139
A16=122.52908564
A17=121.26989442
A18=118.57664488
A19=121.22848482
A20=119.70958789
A21=119.91083724
A22=119.46928428
A23=119.44430407
A24=115.06378607
A25=109.86807712
A26=120.38887613
D1=-0.03390275
D2=0.03929922
D3=-0.0330486
D4=179.99439752
D5=-179.92495427
D6=-179.95562135
D7=-179.93123515
D8=179.59568513
D9=-179.73557368
D10=179.08481868
D11=1.187138
D12=-0.55343503
D13=178.78170643
D14=-66.12580276
D15=114.03769341
D16=-179.71095246
D17=-0.76452602
D18=179.37507628
D19=0.38629443
D20=179.68744154
D21=179.93981235
D22=179.93548529
D23=-178.26527403
D24=174.85766219
D25=-1.84305651

X = Br

Conformer A

Final structure in terms of initial Z-matrix:
C
C,1,B1
C,2,B2,1,A1
C,3,B3,2,A2,1,D1,0
C,4,B4,3,A3,2,D2,0
C,5,B5,4,A4,3,D3,0
H,1,B6,2,A5,3,D4,0
H,2,B7,1,A6,6,D5,0
H,5,B8,4,A7,3,D6,0
H,6,B9,5,A8,4,D7,0
O,4,B10,3,A9,2,D8,0
C,3,B11,2,A10,1,D9,0
C,12,B12,3,A11,2,D10,0
C,13,B13,12,A12,3,D11,0
O,12,B14,3,A13,2,D12,0
C,14,B15,13,A14,12,D13,0
C,16,B16,14,A15,13,D14,0
C,16,B17,14,A16,13,D15,0
C,17,B18,16,A17,14,D16,0
H,17,B19,16,A18,14,D17,0
C,18,B20,16,A19,14,D18,0
C,21,B21,18,A20,16,D19,0
H,19,B22,17,A21,16,D20,0
H,21,B23,18,A22,16,D21,0
H,22,B24,21,A23,18,D22,0
O,13,B25,12,A24,3,D23,0
H,26,B26,13,A25,12,D24,0
Br,18,B27,16,A26,14,D25,0
Variables:
B1=1.38078019
B2=1.40761292
B3=1.40239019
B4=1.39952115
B5=1.38320869
B6=1.08340178
B7=1.08322564
B8=1.08276065
B9=1.08399388
B10=1.35920645
B11=1.4582386
B12=1.46132364
B13=1.35854855
B14=1.23490846
B15=1.47574935
B16=1.40314077
B17=1.4036523
B18=1.38923249
B19=1.08276889
B20=1.39198601
B21=1.39219257
B22=1.08343868
B23=1.0821718
B24=1.0839299
B25=1.35193923
B26=0.97656061
B27=1.91847712
A1=120.32279302
A2=118.7969
A3=121.20508547
A4=118.86085872
A5=120.2271412
A6=121.75866938

A7=119.11908544
A8=119.37224915
A9=122.03269431
A10=122.19933637
A11=115.28640191
A12=121.77416046
A13=125.67431264
A14=125.35351731
A15=118.68592228
A16=123.50116533
A17=121.36287021
A18=118.49817619
A19=121.19522684
A20=119.78803677
A21=119.84750938
A22=119.67705676
A23=119.45285889
A24=115.64745164
A25=104.5436264
A26=121.44905368
D1=0.09891844
D2=-0.12640166
D3=0.08692578
D4=179.98504785
D5=179.96684422
D6=179.91637897
D7=179.90146475
D8=-179.72248863
D9=179.97691737
D10=-179.64289539
D11=0.44795663
D12=-0.19311474
D13=-179.69896313
D14=54.14264789
D15=-125.96086423
D16=178.77172098
D17=-1.07448401
D18=-178.58629468
D19=-0.50389343
D20=-179.67717363
D21=-179.73490239
D22=-179.96105352
D23=179.28727505
D24=-0.92495036
D25=4.28608737

Conformer D

Final structure in terms of initial Z-matrix:
C
C,1,B1
C,2,B2,1,A1
C,3,B3,2,A2,1,D1,0
C,4,B4,3,A3,2,D2,0
C,5,B5,4,A4,3,D3,0
H,1,B6,2,A5,3,D4,0
H,2,B7,1,A6,6,D5,0
H,5,B8,4,A7,3,D6,0
H,6,B9,5,A8,4,D7,0
O,4,B10,3,A9,2,D8,0
C,3,B11,2,A10,1,D9,0
C,12,B12,3,A11,2,D10,0
C,13,B13,12,A12,3,D11,0
O,12,B14,3,A13,2,D12,0
C,14,B15,13,A14,12,D13,0
C,16,B16,14,A15,13,D14,0
C,16,B17,14,A16,13,D15,0
C,17,B18,16,A17,14,D16,0
H,17,B19,16,A18,14,D17,0
C,18,B20,16,A19,14,D18,0
C,21,B21,18,A20,16,D19,0
H,19,B22,17,A21,16,D20,0
H,21,B23,18,A22,16,D21,0
H,22,B24,21,A23,18,D22,0
O,13,B25,12,A24,3,D23,0
H,26,B26,13,A25,12,D24,0
Br,18,B27,16,A26,14,D25,0
Variables:
B1=1.38208547
B2=1.40628781
B3=1.3991519
B4=1.3987034
B5=1.38401242
B6=1.08350566
B7=1.08328061
B8=1.08293125
B9=1.08403925
B10=1.36352327
B11=1.47556283
B12=1.47366195
B13=1.35711961
B14=1.22155871
B15=1.48303806
B16=1.40363734
B17=1.40349612
B18=1.39146253
B19=1.08431723
B20=1.39209621
B21=1.39297225
B22=1.08338566
B23=1.08222119
B24=1.08388336
B25=1.35876866
B26=0.96526763
B27=1.9140023
A1=120.55814204
A2=118.44684188
A3=121.54536764
A4=118.83442715
A5=120.20207636
A6=121.79608501
A7=119.18770203
A8=119.43020465
A9=121.73640581
A10=120.95432854
A11=113.6373026
A12=121.25548923

A13=123.62772934
A14=124.01531991
A15=119.19519337
A16=122.87213989
A17=121.2705297
A18=118.55353406
A19=121.20548757
A20=119.69088568
A21=119.92129007
A22=119.77153209
A23=119.40716908
A24=115.13134166
A25=109.84534788
A26=120.59939184
D1=0.06188735
D2=-0.10116758
D3=0.08733706
D4=-179.99106646
D5=179.97802135
D6=179.97246089
D7=179.9165649
D8=-179.68186782
D9=179.83819806
D10=-179.17405379
D11=-0.89600954
D12=0.53445797
D13=-178.74442775
D14=69.54431027
D15=-110.61157644
D16=179.66384089
D17=0.53353587
D18=-179.40609357
D19=-0.30993223
D20=-179.70562736
D21=-179.92376054
D22=-179.94956661
D23=178.50909856
D24=-175.52311342
D25=1.78061165
